# Methyl-Cyclohexane Methanol (MCHM) Isomer-Dependent Binding on Amorphous Carbon Surfaces

**DOI:** 10.3390/molecules26113411

**Published:** 2021-06-04

**Authors:** William A. Alexander

**Affiliations:** Department of Chemistry, The University of Memphis, Memphis, TN 38152, USA; w.alexander@memphis.edu

**Keywords:** environmental interfaces, desorption kinetics, intermolecular interactions, temperature effects, molecular mechanics, chemical spills, drinking water, odorants

## Abstract

In January 2014, over 10,000 gallons of methyl-cyclohexane methanol (MCHM) leaked into the Elk River in West Virginia, in a chemical spill incident that contaminated a large portion of the state’s water supply and left over 300,000 residents without clean water for many days and weeks. Initial efforts to remove MCHM at the treatment plant centered on the use of granulated activated carbon (GAC), which removed some of the chemical from the water, but MCHM levels were not lowered to a “non-detect” status until well after the chemical plume had moved downstream of the intake. Months later, MCHM was again detected at the outflow (but not the inflow) at the water treatment facility, necessitating the full and costly replacement of all GAC in the facility. The purpose of this study is to investigate the hypothesis that preferential absorbance of one of the two MCHM isomers, coupled with seasonal variations in water temperature, explain this contrary observation. Calculated intermolecular potentials between ovalene (a large planar polycyclic aromatic hydrocarbon) and the MCHM isomers were compared to physisorption potentials of MCHM onto an amorphous carbon model. While a molecular mechanics (MM) force field predicts no difference in the average interaction potentials between the *cis*- and *trans*-MCHM with the planar ovalene structure, MM predicts that the *trans* isomer binds stronger than the *cis* isomer to the amorphous carbon surface. Semi-empirical and density functional theory also predict stronger binding of *trans*-MCHM on both the planar and amorphous surfaces. The differences in the isomer binding strengths on amorphous carbon imply preferential absorbance of the *trans* isomer onto activated charcoal filter media. Considering seasonal water temperatures, simple Arrhenius kinetics arguments based on these predicted binding energies help explain the environmental observations of MCHM leeching from the GAC filters months after the spill. Overall, this work shows the important implications that can arise from detailed interfacial chemistry investigations.

## 1. Introduction

Many aspects of interfacial chemistry are subtle and detailed [1,2,3,4], but they can have important implications for human health and environmental conservation [4,5,6], ranging from the vital transport of breathing gases across the lung surfactant layer [4] to the potentially deleterious effects of gas reactions on the surface of smoggy aerosols [5,6,7,8,9] and dusty grains [10,11] in the atmosphere. We likely associate interfacial chemistry with being particularly important in heterogenous catalysis processes, which have been the center of industrial advancement over many decades [12,13]. Herein, we investigate a seemingly small facet of an interfacial problem that was at the nexus of concerns regarding industrial malfeasance, human health, and environmental impacts following what a former US National Science Foundation division program director characterized as “one of the largest human-made environmental disasters in this century” [14].

**Figure 1:**
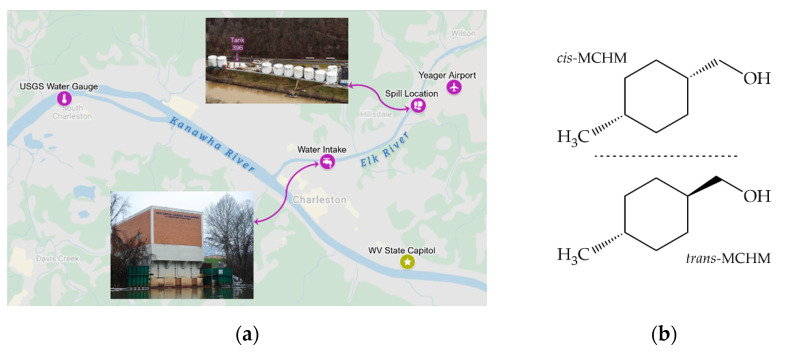
(**a**) Locations in the Charleston, WV area relevant to this study. Base map from Google Maps, inset images from Reference [17]; (**b**) Isomers of 4-methyl-cyclohexane methanol, the major component of the fluid mixture spilled into the Elk River.

On 9 January 2014, residents in WV’s Kanawha Valley awoke anticipating a reprieve from a bitter cold polar vortex that had plunged temperatures from an unseasonably warm 10 to −20 °C within the same day [15,16]. After about 48 h of the chill, as fast-frozen pipes began to thaw, some residents fearing their plumbing would break allowed their faucets to drip and leak what would soon be contaminated water. Little did they know that the frost jack effect was also working its destructive forces on the already weakened floor of a chemical storage tank—number 396—situated on the banks of the Elk River [17], just 1.5 miles upstream from the single drinking water intake [18] that serviced all of their homes and businesses (see Figure 1a). Tank 396 contained a mixture of compounds used as coal frothing agents—surfactants designed to foam and hold onto small coal particles at the surface of coal floatation vessels [19,20]; this allows dirt and rock to easily separate from the high-value coal fines. Two holes in the bottom of Tank 396 allowed passage of over 10,000 gallons of the coal washing mixture to escape within ~8 h—mostly during the early morning hours of 9 January [17,21]. Residents smelled and complained of a heavy licorice odor during their breakfast plans and morning commutes [17]. The main component in Tank 396 was 4-methyl cyclohexane methanol, or MCHM (see Figure 1b). MCHM is an oily compound with a higher boiling point (~200 °C) than water [22] and a sweet licorice odor that our human noses can detect at about 100-fold lower concentrations than the best analytical methods that were available at the time [23,24,25,26]. Beyond the ruptured tank bottom, multiple secondary levels of containment failed at the poorly maintained facility, allowing a nearly unimpeded flow into the Elk River [17].

Because of the warning smell, personnel from the water treatment facility were able to assess the spill in time to make quick decisions about the contaminated water drifting their way [17]. Unfortunately, they were hindered in their decision-making process by the lack of data available regarding the physicochemical properties, toxicity, and mitigation strategies for the MCHM mixture [17,27,28]. While the MCHM mixture is used regionally in quite high quantities, it was still classified as a low volume production chemical, and, like thousands of chemicals in commerce today, MCHM is grandfathered into the Toxic Substances Control Act of 1976 [28,29,30,31,32]. Based on limited guidance form the chemical facility, the Kanawha Valley Treatment Plant’s water intake was never closed in fear of depressurizing the system and losing firefighting capabilities [17]. Contaminated water entered the facility, where operators believed turning up the potassium permanganate and using copious granulated activated carbon (GAC) would sufficiently remove the spilled components from the drinking water [17]. These mitigation efforts were insufficient, and contaminated water was distributed across the 2200 miles of plumbing in the single distribution network that serves over 300,000 people [27,28]. Within a few hours, and over the next few days, residents were turning up in local emergency rooms with exposure-related symptoms [28]. A “Do Not Use” order was enacted by the water company and the governor [33,34]. Residents and businesses across nine counties shut off their taps and waited for a solution [17,28].

The treatment strategies were largely ineffective [17]. Sampling data from 5:00 p.m. on 9 January showed an MCHM concentration at the plant intake of 13.7 ppm, which the treatment facility was only able to decrease to 4.6 ppm in samples of the finished water [17]. A day later, samples showed that MCHM levels of 1.04–3.35 ppm at the intake were only being reduced to 1.02–2.4 ppm [17]. Eventually, sample levels were able to consistently maintain <2 ppb levels of MCHM in the drinking water systems, however, issues regularly manifested out in the distribution system. School children returning from long weekends would smell licorice from the taps, weeks after flushing guidance, prompting school cancellations [35,36,37]. One reason for these pop-up incidents is that MCHM had previously seeped into the polymer plumbing during the long contact time while the “Do Not Use” order was in effect. Once the lines were flushed and filled with clean water, the now-reversed concentration gradient across the polymer surface made the MCHM leech back into the water. This was particularly bad in schools where personnel and students would be absent for a long weekend, allowing the stagnant water to build up these MCHM concentrations for many days [38]. As improved spring weather arrived, and surface temperatures increased, however, it was announced on 24 March that MCHM was again being detected (in samples collected on 21 and 22 January) at the finished water output of the treatment facility, but not at the intake [39]. It seemed the GAC was releasing adsorbed MCHM into the water system, necessitating the full-scale removal and replacement of all GAC filters, at a large cost [40].

A simple question motivates this study: Why did the GAC not work better to adsorb the MCHM and keep it out of people’s premise plumbing? The answers may lie in the varying behavior of MCHM *cis* and *trans* isomers and, importantly, how these isomers participate in interfacial interactions with environmentally relevant interfaces. In this study, we examine the isomeric dependence on the physisorption potential between MCHM and carbonaceous materials using computational molecular modelling approaches. The physisorption potential is predicted using computation at the molecular mechanic force field, semi-empirical, and density functional levels of theory. We consider MCHM in contact with both planar and amorphous surfaces, with surface-site sampling performed using molecular dynamics techniques. We then use the predicted differences in surface binding in kinetic analysis to determine whether seasonal surface water temperatures explain the observed MCHM concentration trends at the treatment facility.

## 2. Results

### 2.1. Computed MCHM Physisorption Potentials with Carbon Surfaces

#### 2.1.1. *cis*- and *trans*-MCHM Plus Ovalene PAH Structures

First, we investigated how *cis*- and *trans*-MCHM physisorb on a model of an ideal flat carbon surface, such as graphene. We employed the polycyclic aromatic hydrocarbon (PAH) ovalene (C_32_H_16_) for this model surface. *cis*- and *trans*-MCHM were allowed to optimize on the ovalene surface to form MCHM:PAH complexes, and a predicted physisorption potential was computed using the MMFF94x force field. Conformational averaged physisorption potentials of 6.2 and 6.1 kcal mol^−1^ were predicted for the *cis*-MCHM:PAH and *trans*-MCHM:PAH complexes, respectively. The energies obtained from these force field computations are not generally considered to be quantitatively accurate, but comparisons can be made between the energies with more confidence. In this case, with the difference between the two complexes of only ~0.1 kcal mol^−1^, the molecular mechanics force field would indicate that on average, the *cis*- and *trans*-MCHM show no difference in physisorption potential on our planar carbon model.

The MCHM:PAH isomer complexes were evaluated at higher levels of theory to confirm whether the MMFF94x predictions would hold. The force field-predicted geometries were optimized with the semi-empirical PM7 Hamiltonian. In addition to PM7, energies were also calculated using the popular density functional theory (DFT) method B3LYP with a modest basis set and inclusion of empirical dispersion (labelled B3LYP-D3 in Table 1) (these methods are further detailed in Section 4.1.1). The intermolecular attraction between MCHM and ovalene is predicted to be over twice as strong with PM7 and DFT (13.9 and 13.5 kcal mol^−1^, respectively, for the *cis*-MCHM:PAH complex) than with MMFF94x (6.2 kcal mol^−1^). Comparing the relative *cis*- and *trans*-MCHM:PAH results indicate that there is in fact a small but significant difference between the physisorption potentials of the *cis-* and *trans*-MCHM isomers with ovalene. The PM7 results indicate that the *trans* isomer should physisorb about 1.9 kcal mol^−1^ stronger than the *cis*. DFT calculations at the PM7 geometries indicate a similar but slightly smaller value, all of which are summarized in Table 1. It seems that some aspects of the attractive potential interaction vary between the *cis*- and *trans*-MCHM isomers interacting with the planar surface, and these subtle effects are not captured by the simple molecular mechanics model.

#### 2.1.2. *cis*- and *trans*-MCHM Plus AC Structures

While the MCHM isomers show no preferential absorbance to the planar surface, GAC and other carbonaceous filter media are not perfect planar structures. We next examine how the *cis*- and *trans*-MCHM physisorb on a more realistic model of these amorphous surfaces. We used as a starting structure, an amorphous carbon model specifically designed with realism in mind, rather than a random model [41]. After equilibration and sampling of this AC slab model, MCHM was allowed to optimize on the surface to form an adsorbed MCHM:AC species. Energy optimization was performed using two different force fields, MMFF94x, and Amber14:EHT within MOE2019. The discrepancy in predicting quantitative energies with MD is apparent in our results. The particular parameters within Amber14:EHT result in predicted physisorption potentials ~4 kcal mol^−1^ stronger than those predicted by MMFF94x. As with the PAH surface, though, we are interested and more confident in the relative energy differences. Both force fields predict similar results: the *trans*-MCHM binds to the AC surface about 2 kcal mol^−1^ stronger. *trans*-MCHM:AC is predicted to be 2.4 (Amber14:EHT) and 1.3 (MMFF94x) kcal mol^−1^ greater than the *cis*-MCHM:AC on average. The true difference in physisorption potential may be even greater, as inspection of individual values showed the Amber14:EHT potentials consistently achieving 3–4 kcal mol^−1^ stronger binding with the *trans* isomer than the *cis*. To further investigate this possibility, the PM7 semi-empirical method was used to calculate the physisorption potential at the Amber14:EHT geometries. The PM7 method predicts even stronger binding to the AC (>10 kcal mol^−1^ stronger than MMFF94x predictions), with the relative energy difference indicating that the *trans* species adsorbs stronger that the *cis* species by 4.7 kcal mol^−1^. Overall, these data imply that the true difference in binding between the two isomer species on amorphous carbon surfaces is likely to be 4 kcal mol^−1^ or greater.

### 2.2. Kinetic Analysis of MCHM Isomer Desorption from Carbon Surfaces

With the prediction that the *trans*-MCHM binds ~4 kcal mol^−1^ stronger (or even more) to AC than *cis*-MCHM, we can examine how this would manifest in the relative desorption kinetics of these two species. Our best estimate for water temperatures is 0–2 °C and ~10 °C for 19 January and 22 March, respectively; we therefore use 273 and 283 K for the relevant kinetic temperatures. Using the Arrhenius equation, MCHM isomers with two different binding energies will desorb at different rates at a particular temperature given by the ratio,

(1) rcisrtrans=e−ΔεkbT,

where Δε is the difference in physisorption potentials (the MCHM-AC binding energy) between the two isomers. This ratio varies with temperature, and as temperature increases the desorption rate of the more tightly bound species “catches” up with the desorption rate of the more loosely bound species. How quickly the rates catch up with each other depends on the magnitude of Δε. In Table 2, we include the results for Δε values between 1.3 and 8 kcal mol^−1^ and from 0 to 30 °C (273–303 K).

## 3. Discussion

### 3.1. Isomeric Differences in MCHM Properties

In the wake of the MCHM spill, much work was carried out to characterize the properties and behavior of MCHM, and to a lesser extent, the other components of the crude MCHM mixture in Tank 396. Various computational models are regularly used in incident response situations as they can predict—almost instantaneously—physicochemical, fate and transport, and biological activity properties [28]. However, it was quickly pointed out by researchers that most quick field-level estimations did not account for differences in isomers [25,27,29,31,42,43]. While there was some variation between groups due to methods and sampling, it was determined that MCHM made up approximately 85% of the spilled liquid, with a *cis*:*trans* ratio of about 1:1.75 [43]. Of particular relevance to examine in this study are experimental and computed estimates of partitioning coefficients, aqueous solubility, and carbon sorption behavior. The octanol–water partitioning coefficient, log *K*_OW_, can be estimated using these programs. EPA’s EPISuite [44] returns a log *K*_OW_ value of 2.55, and ARChem’s SPARC [45] predicts the same value to be 2.43 [46]. Other programs may report these log *K*_OW_ values as the more specific log *P* or log *D*, which vary depending on whether the solute ionizes in solution. As MCHM does not ionize in water to any appreciable extent unless the pH is very low (MCHM’s pKa <2), log *P* and log *D* should not significantly differ at neutral pH. At a pH of 7.4, ACD/Labs^®^’ Percepta^®^ [47] and ChemAxon’s Chemicalize [48] platforms predict log *P* (log *D*) values 2.36 and 1.88 (2.24 and 1.88), respectively. None of these models consider the isomeric nature of the MCHM molecule. Experimentally, log *K*_OW_ was determined using reverse-phase HPLC to be 2.35 and 2.46 for the *cis*- and *trans*-MCHM isomers, respectively [43].

While these estimates are not horrible in comparison with the experiment, larger discrepancies are seen in estimates of water solubility. EPISuite predicts a water solubility value of 2024 mg L^−1^, while Chemicalize predicts 969 mg L^−1^. Within SPARC and COSMO-RS [49], a particular temperature can be specified. SPARC predicts solubility values of 2900 and 1340 mg L^−1^ at 21 and 5 °C, respectively, while COSMO-RS gives 2575 and 2660 mg L^−1^ at the same temperatures [46]. The two methods predict contrary temperature trends. Experimentally, aqueous solubilities at 23 °C were determined using the slow-stir method to 2600 and 2020 mg L^−1^ for the *cis*- and *trans*-MCHM, respectively [43]. At 4 °C, the values are 2780 and 2240 mg L^−1^ [43]. These aqueous solubilities reported in Ref. [43] are based on the experimentally determined value for a 50:50 mole fraction mixture, and as such are one-half of the value expected for pure compounds of *cis*- or *trans*-MCHM. To facilitate direct comparison with the other predictions in the text, we have extrapolated this result directly, so there may be small discrepancies to the true solubility of the pure isomeric compounds. The experimental results clearly show large solubility differences between the isomers, which are not able to be predicted from the algorithmic models. Much of the difference between solubility and partitioning behavior can be explained by considering the specific dipole character of the individual isomers. Of two similar sized and charged compounds, increased aqueous solubility and increased partitioning out of the octanol phase and into the water phase should be seen for the compound with the greater dipole moment. A detailed quantum chemical study revealed that to fully appreciate this difference in the MCHM isomers, both Boltzmann averaging of the low-lying conformers, as well as solvation effects had to be treated [42]. Computed dipole moments in the gas phase predicted the *cis* isomer to be more polar; when solvated, the *trans* species is predicted to be more polar, which is consistent with the experimental results [43].

### 3.2. MCHM Sorption and Desorption

Experimental groups have also examined MCHM sorption and desorption in the lab on polymers [38], coal [19,20,50], activated carbons [31,50], and riverine sediment [31]. Studies of polymer plumbing were mentioned earlier in context of the school closings. They showed that MCHM readily sorbed and desorbed from polyethylene and epoxy pipe linings, and even cause polyurethane to swell and deform [38]. Taken together, the coal and activated carbon experimental results indicate that while GAC readily sorbs MCHM, it also allows significant desorption [31,38,50]; on the other hand, while coal does not sorb as much MCHM, the fraction that does sorb is held tightly and does not readily desorb [19,20,31,50]. Only one of these studies examined the specific sorption differences in light of the specific isomers [31]. Weidhaas and co-workers observed an increase in the MCHM *cis*/*trans* ratio in solution after allowing sorption onto powdered activated carbon [31]. An even larger increase in the ratio was seen in laboratory work on adsorption to sediments from the Elk River [31]; major components of these sediments are carbonate- and silica-based silts and clays, inferring that the isomeric differences in sorption and desorption could be even greater in other filter media, e.g., sand filters. 

**Figure 2:**
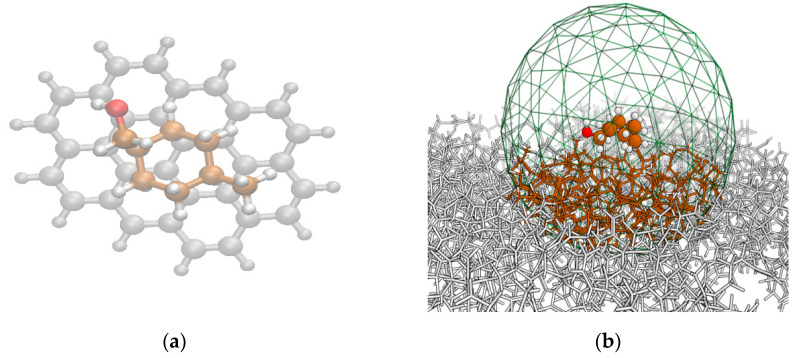
Planar and amorphous carbon surface models used in this study. (**a**) Starting structure of *cis*-MCHM on ovalene PAH. In this view, it can be seen that the ovalene is just large enough to accommodate the maximum footprint of the MCHM molecule. (**b**) Minimum structure of *cis*-MCHM adsorption site on an amorphous carbon surface. The green mesh indicates the 12 Å boundary for building the cluster model. Atoms (colored) within the mesh are subject to higher level computation using the PM7 semi-empirical method (see details in Section 4.1.3 of the text).

Our calculations support Weidhaas’s observations. Because the *trans*-MCHM species adsorbs to AC with a substantially larger physisorption potential (by ~4 kcal mol^−1^ or more) than the *cis*, the *trans* will preferentially adsorb on the surface of amorphous carbons. The molecular mechanics predictions indicate that the MCHM binds with a strength comparable to a strong hydrogen bond, with predictions of about 7.2 and 11.1 kcal mol^−1^ for the MMFF94x and Amber14:EHT force fields, respectively; this is not entirely surprising based on the presence of an alcohol group. What is more surprising is that the *trans* species binds with a significantly greater higher physisorption potential (8.5 and 13.4 kcal mol^−1^, for the same force fields) than the *cis*. Even though it is on the opposite end of the molecule and separated from the hydroxyl group with a nearly-free rotatable methylene unit, the specific orientation of the *cis*-methyl group must still sterically interfere with the MCHM’s optimal orientation in a way that it does not when it is in the *trans* position. In ongoing work in our lab, we are pursuing higher level theory to confirm these energetics using a cluster model of the AC slab. In Figure 2b, the green mesh indicates a 12 Å interaction sphere from the MCHM center of mass that is scooped out from the surface and capped with hydrogens. This smaller size model (circa 600 atoms vs. 6000 in the slab) can be approached with higher levels theory. These results will be reported in different context in the future, but here we can report results of semi-empirical calculations using the PM7 Hamiltonian in Gaussian16 [51]. Using the cluster model, single-point PM7 energies computed at the Amber14:EHT geometries predict that the binding of MCHM may actually be much stronger than the molecular mechanics models predict, in the range of 15–23 kcal mol^−1^ (average of 18.6 kcal mol^−1^) for the *cis*-MCHM species and a range of 20–28 kcal mol^−1^ (average of 23.2 kcal mol^−1^) for the *trans*-MCHM species. Additional preliminary PM7 energy computations at the PM7-optimized cluster geometry indicate that MCHM would physisorb even more strongly (~25% deeper, e.g., 19–33 kcal mol^−1^ rather than 15–28 kcal mol^−1^). Next, we will discuss how these energy differences manifest in predicted desorption kinetic rates.

### 3.3. Kinetic Analysis of MCHM Isomer Desorption from Carbon Surfaces

**Figure 3:**
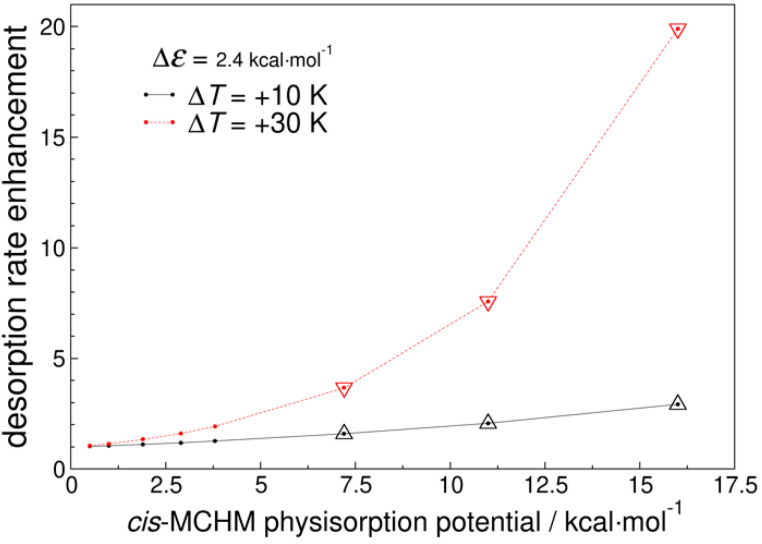
Enhancement in the predicted total desorption rate for MCHM from amorphous carbon as a function of hypothetical *cis*-MCHM physisorption potentials for temperature increases of 10 and 30 K. Enhancement is relative to the rate at 273 K. Results are plotted for the case where the difference in physisorption potentials for *cis*- and *trans*-MCHM is 2.4 kcal mol^−1^; the trend is similar for any value of Δε. The triangles highlight relevant values predicted by the molecular mechanics and semi-empirical predictions detailed in the text.

We have three pieces of information that can be used to infer the relative rate of desorption for different isomers of MCHM: (1) the MCHM-AC physisorption potential, $E_{a}$; (2) differences in physisorption potential between the *cis*- and *trans*-MCHM, Δε; and (3) a change in seasonal water temperatures, ∆*T*. There remain uncertainties about the exact quantitative values of the physisorption potentials, but we now have good estimations of the reasonable ranges. Table 2 displays the relative rates of *cis*:*trans* desorption kinetics at specific temperatures as a function of the difference between their physisorption potentials. The actual desorption rates are also proportional to the actual physisorption potential (as opposed to the difference between the isomers). In this way, the physisorption potential acts as the activation energy barrier for desorption, and the rate is proportional to the population of sorbed molecules that have a larger kinetic energy than the physisorption potential. This high-energy fraction,$\;{\mathrm{frac}}_{hi-\varepsilon }$, of the population is obtained from integrating the Maxwell–Boltzmann distribution of energies,

(2) frachi−ε=∫Ea∞2π(πkbT)32ε12e−εkbTdε,

where $E_{a}$ here is the physisorption potential and the integral is taken over the possible kinetic energies, ε. At a particular temperature, the fraction of molecules in the distribution with sufficient energy to overcome the physisorption barrier decreases as $E_{a}$ increases. Conversely, when a particular $E_{a}$ is set, the fraction of sufficiently high-energy molecules increases as *T* is increased. We can combine these fractions with the relative rate data in Table 2 to arrive at an enhancement factor value, EF, that indicates how the total desorption rate is enhanced when the temperature is increased, and as a function of the activation energy to desorption. We plot in Figure 3 the EF values for temperature changes of 10 and 30 K over a range of hypothetical *cis*-MCHM physisorption potential values for Δε = 2.4 kcal mol^−1^. This energy difference corresponds to that predicted by the Amber14:EHT force field; the trend is similar for any value of Δε. All these values are reported relative to the *cis*-MCHM desorption rate at 0 °C. More details of this derivation are in the Appendix A.

Inspection of Figure 3 reveals that an increase of only 10 degrees in the water temperature can have a substantial impact on the desorption rates. Taking the MMFF94x predicted *cis*-MCHM-AC binding energy of 7.2 kcal mol^−1^, the total desorption rate at 10 °C is 59% higher than at 0 °C. This EF continues to increase as the physisorption potential increases, such that with an $E_{a}$ value of 16 kcal mol^−1^, there is approximately 3 times the desorption rate at 10 °C as would be seen at 0 °C. Furthermore, also plotted are comparable EF values for a 30 °C temperature, indicative of peak summer water temperatures on the Elk River. The EF is quite impressive, with expected ~3.7-, 7.6-, and 20-fold desorption rate increases compared to that at 0 °C, based on the 7.2, 11, and 16 kcal mol^−1^ $E_{a}$ values in the ranges predicted by the MMFF94x, Amber14:EHT, and PM7 computations, respectively.

Digging slightly deeper into the EF values in Figure 3, let us consider the maximal plotted values at each temperature and think about what these values really mean. (For simplicity, we assume a 1:1 *cis*:*trans* ratio throughout this analysis.) For the ∆*T* = +10K EF of ~3 (with a representative value for $E_{a}$ = 16 kcal mol^−1^), this means for every 1000 MCHM molecules that would desorb at 0 °C, 3000 will desorb at 10 °C in the same timeframe, but the total MCHM is not the whole story. Of those 1000 MCHM molecules desorbing at 0 °C, 991 would be the *cis* isomer and only 9 would be the *trans* isomer. Once we move to 10 °C, three times as many molecules would desorb, but the individual isomer ratios change. The 3000 desorbing MCHM molecules consist of 2967 *cis* and 33 *trans* species. The “isomer specific” EF would then be 2.99 for *cis*-, but 3.65 for *trans*-MCHM. The same analysis gives 20,000 MCHM molecules at 30 °C relative to the 1000 at 0 °C. Of these, 19,706 are *cis* and 294 are *trans*, yielding isomer specific EF values of 19.9 for the *cis*, but 32.7 for the *trans*.

### 3.4. Odor Implications in Drinking Water

Thinking about the isomer-specific EF values is especially important for the case of MCHM within the overall context of this investigation. The MCHM spill contaminated residential drinking water supplies with what was a highly odorous compound. In odor threshold testing, it was determined that not only is there a slightly different perceived smell between the *cis* and *trans* isomers, but also different odor detection limits. “Licorice odor” was always the headline and the warning sign during and after the MCHM spill. However, it is only the *trans*-MCHM species that is strongly associated with the infamous telltale sweet licorice scent [25]. The *cis*-MCHM has more subtle scent characteristics, with perceptions ranging from fruit to mint to nail polish [25]. It is also the *trans* species which the human nose is uniquely sensitive to. The odor threshold concentrations (OTC) for the *trans*-MCHM isomer was determined to be 0.060 ppb-v in air, while the *cis*-MCHM threshold concentration was 120 ppb-v in air [25], meaning the human nose is expected to be ~2000 times more sensitive to the *trans*-MCHM isomer. An even more extreme difference was seen based on participant age, with data suggesting that subjects <50 years old (min age 20) were over 100,000 times more sensitive to detection of the *trans* species compared to the *cis* [25]. From our kinetic analysis, we know that the *trans* species is the minority component of MCHM desorption from carbonaceous sorbent material, but with it being perceivable at a 2000× lower concentration, the increase in odor as a function of temperature should be striking. Much smaller changes in the *trans*-MCHM concentrations could be enough to be detected through smell than with the *cis*-MCHM concentrations. Taste and odor problems in water supplies can drive fear and engender outrage, prompting the public to demand solutions to what is thought to be a risky situation [52]. Even if the contaminant concentration is safe from a health perspective, odor and taste perception are important consumer issues for drinking water operations. Our analysis herein shows that partitioning behavior at the carbon filter interface may have played a significant role in the on-going drinking water quality issues following the MCHM spill in 2014.

## 4. Materials and Methods

### 4.1. Molecular Modelling

**Figure 4:**
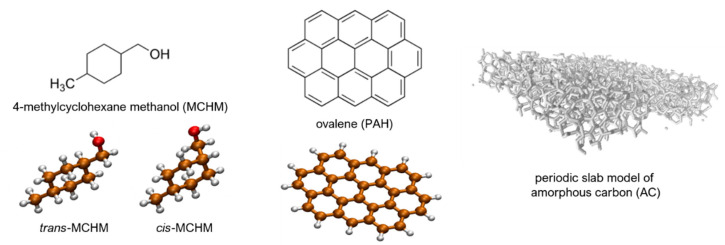
Structures used in this study.

This section provides more details of the models used in our molecular modelling approach. Structures mentioned in the proceeding sections are included in Figure 4 for visual reference.

#### 4.1.1. *cis*- and *trans*-MCHM Plus Ovalene PAH Structures

Ovalene is the smallest symmetrical planar polycyclic aromatic hydrocarbon (PAH) that is large enough in both planar dimensions to allow an MCHM molecule to “lay flat” on the center and still have about one benzene unit in either direction before the edge. Starting structures for *cis*- and *trans*-MCHM interacting with the ovalene (C_32_H_14_) were arranged by hand to ensure relatively close contact with the center of the ovalene molecule (see Figure 2a), minimizing edge effects. Previous work showed that both MCHM isomers can adopt a plethora of low-energy local minima [42], so it is important to sample the various conformations of MCHM as well as the relative orientations between the MCHM and the ovalene “surface.” Both aspects were accounted for using the LowModeMD search program in the MOE software package [53]. The LowModeMD search algorithm in MOE generates conformations using short (~1 ps) molecular dynamics (MD) simulations at constant temperature, followed by all-atom geometry optimizations [54]. By default, the energy minimization technique in MOE utilizes the MMFF94x force field [55] and incorporates a distance-dependent solvation effect with a dielectric constant of 80 for the “exterior” or solvent-exposed parts of the system. MMFF94x is a general all-atom force field parameterized for small organic molecules, which incorporates improvements over the initial MMFF94 (Merck molecular force field) (for instance, MMFF94x predicts planar conjugated nitrogens, while MMFF94 predicts tetrahedral nitrogen conjugation) [55]. MCHM:PAH complexes with relative energies higher than 7.0 kcal mol^−1^ from the lowest energy conformer were discarded. These searches provided 120 conformers for the *cis*-MCHM:PAH complex from 800 poses, and 138 conformers for the *trans*-MCHM:PAH from 809 poses. Intermolecular energies were determined by taking the MMFF94x energy computed for the MCHM:PAH complexes and subtracting that computed for a lone ovalene. Then, the average of the five lowest MCHM conformers (reported previously, see [42,43]) was subtracted to obtain an apparent physisorption potential that is relative to the first few low-lying conformers. 

The MCHM:PAH isomer complexes were also evaluated at higher levels of theory. The MMFF94x force field-predicted geometries were further optimized with the semi-empirical PM7 Hamiltonian in Gaussian16. Of the initial 120 *cis*- and 138 *trans*-MCHM:PAH complexes, 114 and 129 structures, respectively, converged to unique geometries. In addition to PM7, energies were calculated using the popular density functional theory (DFT) method B3LYP with a modest basis set, 6–31G(d), as implemented in Gaussian16. Empirical dispersion was included using the D3 version of Grimme’s dispersion method with Becke–Johnson damping [56]. The DFT energies were calculated at the PM7-optimized geometries. For both PM7 and DFT computations, the physisorption potential was taken as the difference in the computed heats of formation between the MCHM:PAH complex and the sum of the lone MCHM molecule and the lone PAH, calculated with the same geometry as was in the optimized complex.

#### 4.1.2. Amorphous Carbon (AC) Model 

We used a previously thermally equilibrated periodic slab model of amorphous carbon as a starting structure [41]. The initial structure was originally developed using a semi-empirical tight binding approach implemented in a canonical Monte Carlo algorithm. An advantage of the model is that it gives a particularly good description of the *sp*, *sp*^2^, and *sp*^3^ hybrid bonds; the resulting structure was also shown to be more accurate to describe Cu, Ag, and Au atom adsorption than a model constructed from randomly generated carbon positions [41]. To prepare a slab model of an amorphous carbon surface, the *z*-dimension of the 5 × 5 × 3 nm periodic box was extended to 25 nm, while the *x*- and *y*-dimensions were maintained with periodic boundary conditions, creating two solid-vacuum interfaces. Dangling bonds were saturated with hydrogens in MOE, and the surface was allowed to equilibrate through a molecular dynamics run in MOE 2013. Molecular dynamics were carried out with NAMD 2.9 [57] at 300 K. An initial 100 ps equilibration run was followed by a 500 ps production run. Molecular geometry and velocity snapshots of the AC slab taken along the production run were saved for later use. The resulting AC surface was then used to study adsorption of MCHM as a surrogate model for GAC and similar materials. It is noted, however, that our AC model is of pure carbon capped with hydrogen. Real GAC and other materials will have some level of oxidized functional groups on the surface as well, and this simple model does not capture effects from those groups.

#### 4.1.3. *cis*- and *trans*-MCHM Plus AC Structures

*AC Slab Model.* Adsorption sites on the central ~3 × 3 nm area of one face of the AC were sampled for each isomer by roughly aligning a subset of the MCHM:PAH conformations with the AC surface. The PAH was aligned with the AC plane, and the PAH removed, leaving the MCHM in a favorable starting orientation. Randomized orientations were selected, and the three lowest MCHM:PAH conformers for each isomer were intentionally included in the sample set. The MCHM isomer was then allowed to minimize to the rigid AC surface using one of two force fields, MMFF94x or Amber14:EHT [58,59], as implemented in MOE2019 [53]. Predicted physisorption potentials for each MCHM:AC were taken as the intermolecular van der Waals interaction energy computed between the MCHM and the AC.

*AC Cluster Model.* Subsequently, the PM7 semi-empirical method was used to calculate the physisorption potential at the Amber14:EHT geometries using a cluster model of the surface. To form the cluster model, all atoms within a 12 Å interaction sphere as measured from the MCHM center of mass were selected (see Figure 2b). Exterior atoms were discarded and dangling bonds were automatically capped with hydrogen within MOE2019; structures that resulted in excess charge or non-singlet multiplicities were discarded. About two-thirds of the Amber14:EHT MCHM:AC geometries remained and were used as input for PM7 computations in Gaussian16. The physisorption potential was taken as the difference in the computed heats of formation between the cluster complex and the sum of the lone MCHM molecule and the lone cluster, calculated at the same geometry as was in the cluster complex. Because several structures were discarded automatically, it is especially important to ensure that the differences between the *cis* (*M* = 18.6, *SD* = 2.4) and *trans* (*M* = 23.2, *SD* = 2.3) isomers are significant and not biased by this sampling. Statistical analysis indicates the differences in the means is significant with the independent two-tailed *t*-test yielding *p* < .001, with a size effect of 2.006 (Cohen’s *d* value).

### 4.2. Surface Water Temperature Estimations

#### 4.2.1. Historical Average Water Temperatures

Ideally, water temperature data from the water treatment plant, and specifically of the GAC filters, would be used in the kinetic analysis herein. Those data were not readily available. Water temperatures *after* the spill were reported as 3.5 ± 0.1 °C and 3.2 ± 0.7 °C on 16 January and 20 January, and 24.6 °C on 18 August [31], a few days and then months after the relevant days. However, historical daily water temperature measurements were found to be available from a USGS gauging station (USGS 03198000) on the Kanawha River in South Charleston, WV, (see Figure 1) only ~4.4 river miles downstream from the water facility intake [60]. These data span the period from 1 April 1950—9 April 1985; other than a gap in coverage from 7 August 1970—1 October 1971, 93% of days have recorded minimum and maximum daily water temperatures. Average historical daily water temperatures were calculated from measurements taken within one week of the two significant dates in the MCHM spill story—9 January and 22 March. Average historical water temperature highs and lows for January 2–16 were 5.58 (407 temps, σ = 2.45, range 0.5–15.0 °C) and 4.21 °C (407 temps, σ = 2.29, range 0.5–10.6 °C), respectively. For March 15–29, average highs and lows were 8.96 °C (420 temps, σ = 1.76, range 4.0–14.5 °C) and 8.46 °C (420 temps, σ = 1.69, range 3.9–14.0 °C), respectively. This shows that historically there should be an expected ~4 °C difference in river water temperatures in the area from 9 January to 22 March. This historic average does not however account for incidental variation in water temperatures due to local weather and microclimate events.

#### 4.2.2. Accounting for Effects of Ambient Air Temperature (i.e., Weather) 

Rather than rely on historical water temperature data, which at best was 23 years old relative to the spill incident, it would be convenient to use ambient atmospheric weather data (i.e., regular weather reports), as these data are available hourly from the nearby Yeager Airport located only 0.6 and 2.2 miles from the spill site and water intake, respectively (see Figure 1) [61]. Attempts to correlate historical water data with the weather reports to produce a *simple* predictive model were not fruitful. It should perhaps be obvious that the high heat capacity of water largely resists the sometimes-quick changes in weather. This is easily seen in the historical water temperature data, which show average daily temperature variations of only 1.08 (2–16 January, σ = 1.14), 0.49 (15–29 March, σ = 0.46), and 1.05 (year round, σ = 0.91). For a specific example of how contrary these measures can be, on 9 January 1966, reported temperatures were 7.8/7.8 °C (hi/lo, water) and 4.4/−3.9/−12.2 °C (hi/avg/lo, air), while on 9 January 1984 those same measures were 2.0/2.0 °C (hi/lo, water) and 15/4.4/−3.3 °C (hi/avg/lo, air). It should also be noted that precipitation events likely shift water temperatures more quickly than air temperature changes, but we make no attempt to correlate/correct for the effects in this study.

While weather may not change water temperatures as fast as air temperatures, our predictions based on historical averages can be hedged up or down based on the unique and specific weather conditions within the days *leading up* to the spill incident (~9 January) and re-appearance of MCHM at the plant (~21 March). Historical weather averages report air temps of ~1.1 °C for Jan, in line with the 9 January 2014 average temperature of 0.97 °C; however, leading up to this date, temperatures were well below average, with average daily temperatures of −6.7, −16.0, and −8.0, for 8, 7, and 6 January, respectively, with a new all-time record low recorded on 7 January of −19.4 °C. These sustained lows would certainly drive down the water temperature, leading us to an estimate of 0–2 °C used in the kinetic analysis. Historical weather averages report air temps of ~8.9 °C for March, and the 22 March 2014 average temperature was close at 8.6 °C. However, while 22 March was at the average temperatures, the days previous were unseasonably hot. Daily average temperatures were reported as 12.7, 14.5, and 3.5 °C, for 21, 20, and 19 March, respectively, with highs of 20.6 and 20 °C on 21 and 19 March, respectively. The sustained higher temperatures would increase the water temperature, lending confidence to estimating water temps above 10 °C used in the kinetic analysis. Finally, we note that the maximum water temperature recorded in the historical data was 35 °C, while temperatures at the gauging station recorded in mid–late summer consistently reached a degree or two above 30 °C. Coupled with the reported ~25 °C water temp for 18 August [31], this gives validation to perform a kinetic analysis at these higher temperatures.

## 5. Environmental Implications and Concluding Remarks

Overall, our findings indicate that MCHM may physisorb to planar carbon surfaces with attractive potentials of around 15 kcal mol^−1^ and may bind even stronger to amorphous/rough carbon surfaces (circa 20–25 kcal mol^−1^). Importantly, however, physisorption is not equal with both isomers. The *trans*-MCHM isomer is predicted to bind to amorphous carbon surfaces ~4 kcal mol^−1^ or more strongly than the *cis*-MCHM isomer. The preferential binding effect is more subtle on planar carbon, such that higher levels of theory than molecular mechanics force fields are needed to sufficiently predict this difference. A kinematic analysis of relative desorption rates taking these differential adsorption strengths of the MCHM isomers into account indicates that an overall enhancement factor of *trans* desorption relative to *cis* could be 20-fold or higher based on a shift from winter-to-summer seasonal water temperatures.

With regard to the aftermath of the 14 January MCHM spill, our data and analysis help to explain the contrary results of MCHM detection in the finished water months after it was undetectable in the source water. Of course, our model is simple and uses ideal, clean surfaces in our computations. Our model does not account for complicating effects found in the field, such as dissolved biomatter, competition with other would-be adsorbates, etc., but the basic concepts still hold. Nevertheless, even this simplistic level of analysis indicates that (1) binding of MCHM to GAC and other carbonaceous filter media is not strong enough for permanent removal from the water system, (2) differences in the isomer physisorption potential would lead to differential sorption and desorption behaviors, and (3) desorption from filter media into the water system should be expected as seasonal temperatures increase. Such insights could have influenced decision making to promulgate mitigation and removal of the contaminated GAC filter media prior to spring temperatures allowing further release of the compound into the drinking water supply. Our data indicate that the Elk River water temperature changes from Jan (~0 °C) to Mar (~10 °C) could have resulted in increasing the total rate of MCHM desorption by as much as a factor of three; had the GAC filters been allowed to continue to desorb MCHM into the heat of the summer (where water temperatures routinely exceed 30 °C), MCHM desorption rates could have increased to over 20 times the initial desorption rates in Jan. Because of the different isomeric desorption rates, as water temperature increases, odor perception of the increased MCHM desorption could see even more drastic scaling than the desorption rates indicate. Beyond the Elk River incident, ongoing development of computational libraries of amorphous surfaces in the author’s lab can be combined with higher level QM/MM calculations to refine the predictions herein, and to make similar predictions for other substances. The general methodology detailed in this study can be applied for future chemicals whose under-characterized or orphan status necessitate estimates of surface binding to many types of amorphous interfaces relevant for filter media, environmental interfaces, premise plumbing, etc.

## Figures and Tables

**Table 1:** Differences in physisorption potentials between MCHM isomers and carbonaceous surfaces.

	Difference in Isomers, *E_trans_—E_cis_* ^1^			
System	MMFF94x	Amber14:EHT	PM7	B3LYP-D3
MCHM:PAH	0.1	-	1.9	1.4 ^2^
MCHM:AC	1.3	2.4	4.7 ^3^	-

^1^ Difference in predicted physisorption potential in kcal mol^−1^. ^2^ B3LYP/6-31G(d) energies, with D3-BJ dispersion, at the PM7 optimized geometries. ^3^ Calculations were carried out on the cluster model with Amber14:EHT geometries (see Figure 2).

**Table 2:** Predicted relative desorption rates of MCHM isomers from amorphous carbon at different temperatures.

$r_{cis}/r_{trans}$				
$\Delta \varepsilon {\;}^{1}\;$	0 °C	10 °C	20 °C	30 °C
1.3	1.29 × 10^1^	1.18 × 10^1^	1.08 × 10^1^	1.00 × 10^1^
2.4	1.06 × 10^2^	9.02 × 10^1^	7.74 × 10^1^	6.70 × 10^1^
4.0	2.40 × 10^3^	1.82 × 10^3^	1.41 × 10^3^	1.11 × 10^3^
8.0	5.53 × 10^6^	3.20 × 10^6^	1.92 × 10^6^	1.19 × 10^6^

^1^ Difference in physisorption potential in kcal mol^−1^.

## Data Availability

Data presented in this study may be made available on request from the corresponding author.

## Sample Availability

Samples of the compounds are not available from the author.

## Appendix A

*Total MCHM Desorption Rate Enhancement Factor*. The amount of *cis*-MCHM that desorbs is proportional to the fraction of *cis* molecules, ${\mathrm{frac}}_{hi-\varepsilon ,cis}$, that have enough energy to overcome a given physisorption potential, $E_{a}$, at a given temperature, *T*; this fraction is obtained by integrating the Maxwell–Boltzmann distribution (Equation (2)) over the $E_{a}\to \infty$ limits. The fraction of *trans*-MCHM molecules that desorb relative to ${\mathrm{frac}}_{hi-\varepsilon ,cis}$ is obtained using the relative rates from the Arrhenius equation (Equation (1)) for a particular temperature, *T*, and given the difference between the isomers’ physisorption potentials, Δε, as

(A1) frachi−ε, trans=frachi−ε, cisrcisrtrans.

The total amount of MCHM desorption at a given set of *T*, $E_{a}$, and Δε values is

(A2) $$\mathrm{total}\;\mathrm{MCHM}\left(T,\;E_{a},\;\Delta \varepsilon \right)=\mathrm{amount}\;\mathrm{of}\;\mathrm{cis}-\mathrm{MCHM}+\mathrm{amount}\;\mathrm{of}\;\mathrm{trans}-\mathrm{MCHM}\;,$$

which is proportional to the computed fractions,

(A3) $$\mathrm{total}\;\mathrm{MCHM}\left(T,\;E_{a},\;\Delta \varepsilon \right)\propto \;{\mathrm{frac}}_{hi-\varepsilon ,\;cis}+{\mathrm{frac}}_{hi-\varepsilon ,\;trans}$$

(A4) ∝ frachi−ε, cis+frachi−ε, cisrcisrtrans.

The enhancement factor plotted in Figure 3 is given by the ratio

(A5) $$\mathrm{EF}=\frac{\mathrm{total}\;\mathrm{MCHM}\left(T_{2},\;E_{a},\;\Delta \varepsilon \right)}{\mathrm{total}\;\mathrm{MCHM}\left(T_{1},\;E_{a},\;\Delta \varepsilon \right)}$$

where all EF values are defined in this paper relative to the total MCHM proportions with *T*_1_ = 273K (0 °C) for each set of $E_{a},\;\Delta \varepsilon$ values. In other words, we set the denominator equal to 1 for each set of $E_{a},\;\Delta \varepsilon$ values at 0 °C. The EF value is then always given in this paper as the enhancement of the total MCHM desorption rate at different temperatures, relative to that at 0 °C. As an example, if we obtain, using Equation (A5), an EF value of 10 at *T*_2_, the total rate of MCHM is 10-fold more at *T*_2_ than what it would be at 0 °C.
